# Fear of progression in patients with acute myocardial infarction: a cross-sectional study

**DOI:** 10.1186/s12912-024-02552-1

**Published:** 2024-11-28

**Authors:** Xinghui Wang, Nan Jiang, Shuoxin Chen, Subinuer Tuerdi, Jiayu Yang, Rong Yan, Li He, Jiajia Wang, Yuewei Li

**Affiliations:** 1 First Affiliated Hospital of Hebei Medical University, Hebei, China; 2 The Second, Bethune Hospital of Jilin University, Jilin, China; 3 School of Nursing, Jilin University, 965 Xinjiang St, Changchun, Jilin 130000 China

**Keywords:** Acute myocardial infarction, Fear of progression, Nursing care, Root cause analysis

## Abstract

**Background:**

Acute myocardial infarction (AMI) is the most serious manifestation of coronary artery disease. At present, existing treatments cannot change the risk factors for the occurrence of the disease, so patients are prone to fear of progression or recurrence, and studies have shown that excessive fear will cause patients to over-examine, mental abnormalities, suicide and other behaviors, increase unnecessary medical care costs and the social medical burden. Thus to investigate the current situation of progression fear in patients with Acute Myocardial Infarction (AMI) admitted to hospital in stable stage and analyze its influencing factors.

**Methods:**

This is a cross-sectional study. In this study, 160 AMI patients admitted to the Department of Cardiology of a 3 A hospital in Changchun from November 2022 to April 2023 were selected as the research sample by a convenient sampling method. Patients completed the general information questionnaire, the Fear of Progression Questionnaire-Short Form (FoP-Q-SF), the Brief Illness Perception Questionnaire (BIPQ), the Medical Coping Modes Questionnaire (MCSQ), the Social Support Rating Scale (SSRS), and the Positive Psychological Questionnaire (PPQ) at the time of stable condition. Using SPSS 25.0 software for data analysis, statistical methods mainly include descriptive statistics, analysis of variance, Pearson correlation analysis and multiple linear regression analysis.

**Results:**

A total of 160 subjects were included, among which the score of simplified fear of progression scale was (33.43 ± 7.09), and the incidence of disorder of fear of progression was 50.60%. The influencing factors included gender (Beta = 0.118, *P*<0.05), disease perception (Beta = 0.445, *P*<0.001), psychological capital (Beta=-0.252, *P*<0.05) and resignation (Beta = 0.167, *P* < 0.05).

**Conclusion:**

The overall level of fear of progression in patients with AMI at the stable stage of hospitalization is above the medium level. Nursing staff should focus on AIM patients suffering from multiple diseases and lacking awareness of their own diseases, and provide personalized health guidance and psychological nursing targeted to improve patients’ rehabilitation, quality of life and FoP level.

## Introduction

Acute myocardial infarction (AMI) is a common cardiac emergency and the most serious manifestation of coronary artery disease [1]. It has been reported that the number of deaths due to acute myocardial infarction in the United States exceeds 2.4 million each year, which is the main cause of morbidity and mortality worldwide [2, 3]. In China, there are about 2.5 million AMI patients, with an increasing incidence and a gradual shift towards younger age groups. This presents a significant challenge to the control and improvement of the prognosis of AMI patients [4, 5]. Despite the enhancement of current treatment methods and the pre-hospital emergency system for acute myocardial infarction, the mortality rate and total hospital stay of patients have been reduced [6, 7]. However, the current treatment methods cannot change the risk factors of disease occurrence, and patients are still prone to adverse cardiovascular events such as in-stent restenosis, recurrent myocardial infarction, and heart failure [8, 9]. As a result, patients are prone to fear of progression or recurrence, which brings huge psychological burden to patients.

Fear of Progression (FoP) is one of the common psychological burdens of patients [10]. It refers to the individual’s fear of everything related to their disease. It is a distinct phenomenon from traditional psychological disorders, such as anxiety and depression. Rather, it is specifically defined as the fear of the biological, social, and psychological consequences associated with the advancement of the disease, or the fear of the disease’s recurrence [11]. Fear of progression is widespread in different diseases, including cancer [12, 13], diabetes [14], rheumatoid arthritis [15] and acute pancreatitis [16]. It has been reported that the current overall FoP of AMI patients in China is at a moderate level [17], with approximately 16.7% of AMI patients exhibiting FoP psychological dysfunction [18]. The experience of surgery, interventional treatment, near-death chest pain and adverse drug reactions can engender a profound sense of helplessness in patients, thereby intensifying their fear of progression [19, 20]. Long-term excessive FoP has been demonstrated to result in a range of adverse effects, including inattention, sleep disturbances, reduced physical endurance and other phenomena. Additionally, it has been observed that patients may engage in avoidance behaviours as a coping mechanism. This has been demonstrated to affect patients’ willingness and effectiveness to participate in early out-of-hospital cardiac rehabilitation, reduce their treatment compliance, hinder their rehabilitation process [21–23], increase the risk of adverse events and negative emotions such as anxiety, depression and post-traumatic stress disorder [24–26], and seriously affect patients’ prognosis and quality of life.

However, the current research on FoP in AMI patients is very limited, most studies only focus on the improvement of anxiety and depression in AMI patients, while ignoring the adverse effects of FoP, and the associated factors remain relatively understudied. Therefore, this paper aims to explore the current state of fear of progression and its influencing factors in AMI patients, with the objective of providing a foundation for the formulation of appropriate and effective intervention strategies to alleviate the fear of progression in AMI patients.

This study will take the stress process theory proposed by Professor Jiang Qianjin as the theoretical basis [27] to guide the discussion on the influencing factors of FoP in AMI patients. It complements and improves the stress and coping theory, which points out that the individual is a multi-factor system. When facing the threat of stressors, the individual will act on the individual through the intermediary factors that can restrict and influence each other, including cognitive evaluation, social support, coping style and personality characteristics, and ultimately affect the disease or health state of the individual. At present, this theory has been applied to explore the influencing factors of FoP in patients with acute pancreatitis and colorectal cancer after surgery [28, 29], but it has not been applied in patients with AMI. In addition, organizational behaviorist Luthans proposed in 2004 [30] that psychological capital is specifically defined as “a positive psychological state shown by an individual in the process of growth and development”. Psychological capital plays a positive guiding role and can be developed and intervened. Therefore, in this study, we regard sudden AMI as a stressor and believe that patients will develop FoP under the influence of cognitive evaluation, coping style, social support and personality characteristics. In this study, the simplified version of the disease perception Questionnaire, the Medical Coping style questionnaire, the social support rating Scale, the psychological capital questionnaire and the general information questionnaire were used to investigate the disease perception, coping style, social support and personality characteristics of AMI patients, in order to explore the influencing factors of FoP in AMI patients.

## Methods

### Study design, setting, and participants

AMI patients admitted to the cardiology department of a tertiary hospital in Changchun, China from November 2022 to April 2023 were selected for the study using convenience sampling method.

Inclusion criteria: (1) aged ≥ 18 years old; (2) Patients met the diagnostic criteria of “Guidelines for Diagnosis and Treatment of Acute Myocardial Infarction” formulated by the Cardiovascular Society of the Chinese Medical Association; (3) Patients with stable condition after PCI surgery, thrombolysis or vasodilator therapy 48–72 h after admission and out of life danger; (4) Patients with informed consent and the ability to read and can normally communicate, as well as to answer questions. Exclusion criteria: (1) AMI patients with cognitive impairment, language impairment, and history of mental illness; (2) AMI patients with complications such as tumor, liver and kidney dysfunction (3) AMI patients receiving psychotherapy.

The sample size was calculated prior to data collection using the G*power software version 3.1 [31]. Using a conventional power estimate of 0.9, with alpha set at 0.05, and an effect size of 0.5, a sample size of 150 participants was required to reduce the probability of Type I and Type II errors. A total of 160 participants were included in the study.

### Evaluation tool and data collection

#### Demographic features

The study includes age, sex, marital status, occupational status, monthly income, educational level, payment method, location of coronary artery infarction, number of coronary artery infarctions, time of onset, treatment method, cardiac function classification, presence of self-help modalities, family history of coronary heart disease, and comorbid chronic comorbidities.

#### Fear of progression questionnaire- short form

The Fear of Progression Questionnaire- Short Form (FoP-Q-SF) was compiled by Mehnert et al. [32] on the basis of the Fear of Progression Questionnaire (FoP-Q), composed of two dimensions of social family and physiological health, with a total of 12 items. Likert 5-level scoring method was adopted for this scale, with 1 point indicating “never” and 5 points indicating “always”. The score range was 12 to 60 points, and the higher the score, the higher the FoP level of patients. If a patient scored 34 points or more, it was considered that the patient’s FoP psychology was above the normal level, indicating FoP psychological dysfunction. In 2015, Wu Qiyun et al. [33]translated it into Chinese, and the Cronbach’s α of this scale was 0.886, which had good internal consistency. In this study, Cronbach’s α of this scale was 0.911.

#### Brief illness perception questionnaire

The Brief Illness Perception Questionnaire (BIPQ) [34] was an assessment of the patients’ feelings and cognition of the disease. It consists of 9 items, among which 5 items are used to evaluate the cognitive symptoms of the disease, 2 items are used to evaluate the emotional symptoms of the disease, 1 item is used to evaluate the patients’ understanding of the disease, and 1 item is an open question for the evaluation of the cause, asking the patients to list the 3 most important causes of the disease. The score ranges from 0 to 80, and the higher the patient’s score, the higher the patient’s negative disease perception. In 2015, it was sinicized and used by Yaqi Mei et al. [35] In this study, Cronbach’s α of this scale was 0.733.

#### Medical coping modes questionnaire

Medical Coping Modes Questionnaire (MCSQ) was compiled by Feifel et al. [36], revised by Jiang Qianjin et al. [37] and introduced into China to evaluate patients’ coping styles with diseases. The scale is divided into three kinds of coping styles: confronce, resignation and avoidance, which represent the fundamental behavioural responses to the threat of disease. In this study, Cronbach’s α of this scale was 0.664.

#### Social support rating scale

The Social Support Rating Scale (SSRS), compiled by Xiao Shuiyuan (1994) [38], has 10 items and is divided into three dimensions, namely subjective support, objective support and utilization of support. Entries are scored on a Likert level 4 or multiple scale, with higher scores indicating higher levels of social support. In this study, Cronbach’s α of this scale was 0.71.

#### Positive psychological questionnaire

The Positive Psychological Questionnaire (PPQ), compiled by scholar Zhang Kuo in 2010 [39], contains 26 items in four dimensions: self-efficacy, hope, optimism and resilience. The positive psychological questionnaire adopts Likert 7-level scoring method, with the reverse scoring questions including 8, 10, 12, 14 and 25. The higher the score of each dimension and total score, the higher the level of positive psychological capital of an individual. Cronbach’s a = 0.955 in this study.

#### Data collection methods and quality control

Before the formal investigation, the purpose, content and significance of the research should be explained to the director of the relevant department and the head nurse of the hospital. After obtaining their consent, the formal investigation should be carried out after familiarising oneself with the department environment and requirements of the department. After the AMI patients were admitted to hospital and their condition was stable, the purpose, content, significance and follow-up time of the study were explained to the AMI patients who met the inclusion criteria. There is no absolute fixed time range for the stable period of the patients with acute myocardial infarction after admission. In this study, this was defined as the period when the patient passed the most dangerous stage after the onset of acute myocardial infarction and the condition was relatively stable, about one week after the onset of the disease. After obtaining the informed consent of the patients, the patients were instructed to sign the informed consent form and promised to keep the relevant information strictly confidential, and the precautions for filling in the questionnaire were explained to the patients. Data were collected in a face-to-face manner, and the questionnaire was completed by the patients independently. For the patients’ questions, the researchers explained them accordingly. For the patients who could not complete the questionnaire independently, the researchers asked questions according to the contents of the questionnaire and assisted them to complete the questionnaire. After the completion of the questionnaire, the questionnaire was collected on the spot, and the options for missing or wrong filling were checked immediately. If there was such a situation, the patient was asked to fill in and modify it accordingly. Finally, the next follow-up time was explained to the patient again and the patient was thanked.

### Statistical analysis

The data analysis was conducted using SPSS 25.0 statistical software, and a p-value of less than 0.05 was considered to indicate a statistically significant difference. Descriptive statistics were used for demographic and disease-related data of patients. Counting data were described by frequency and percentage. Measurement data that conformed to a normal distribution or an approximate normal distribution were described by mean and standard deviation, whereas non-normal distribution was described by median and quartile.

In the single factor analysis, independent sample test or single factor analysis of variance were used when the data were normal or approximately normal distribution with homogeneous variance; otherwise, non-parametric test was used to analyze the influence of different demographic data and disease-related data on FoP levels in patients with acute myocardial infarction. pearson correlation analysis was used to analyze the relationship between FoP level and disease perception, social support, coping style and psychological capital. The index of *P* < 0.05 in the single factor analysis was included in the multiple linear regression model for multiple linear regression analysis to analyze the influencing factors of FoP. SPSS 25.0 was used for data analysis, statistical methods mainly include descriptive statistics, analysis of variance, Pearson correlation analysis and multiple linear regression analysis.

### Ethical approval

This study has been reviewed by the Nursing Ethics Committee of Jilin University (ethics number: 20221103309), and registered in the Chinese Clinical Trial Registry (registration number: ChiCTR2300075568). Before the research is carried out, the participants are fully informed of the purpose, significance and content of the research. During the interview, the participants are allowed to quit midway. They are assured that their personal information will be kept strictly confidential, and they will be invited to complete the questionnaire survey after signing the informed consent form.

## Results

### Characteristics of the participants

A total of 160 patients with AMI were included in this study, with an mean age of (57.81 ± 11.01) years, of which 75 patients were over 60 years old, accounting for 46.9% (Tables 1 and 2).

**Table 1:** Demographic data of patients with acute myocardial infarction (*n* = 160)

Variables	*n*	Percentage(%)
**Gender**		
Male	123	76.90
Female	37	23.10
**Age(year)**		
<45	25	15.60
45 ~ 60	60	37.50
>60	75	46.90
**Marital status**		
Married	148	92.50
Spinsterhood	5	3.10
Divorce/separated	5	3.10
Widowed	2	1.30
**Employment**		
Employed	40	25.00
No job/resignation	60	37.50
Retired	41	25.60
Farmer	19	11.90
**Monthly income**		
≤ 1000 yuan	48	30.00
1000–3000 yuan	60	37.50
3000–5000 yuan	39	24.40
≥ 5000 yuan	13	8.10
**Education**		
Primary and below	25	15.60
Junior high school	60	37.50
Senior high school	63	39.40
University and above	12	7.50
**Payment**		
Medical insurance	153	95.60
Self-financed or other	7	4.40

**Table 2:** Disease related data of patients with acute myocardial infarction (*n* = 160)

Variables	*n*	Percentage(%)
**Type of AMI**		
STE-MI	97	60.60
Non-st elevation type	63	39.40
**Number of coronary artery infarcts**		
Single lesion	90	56.30
Multiple lesion	70	43.80
**Treatment method**		
Drug Conservative therapeutic	32	20.00
Interventional Surgery	128	80.00
**Onset time**		
7: 00–18–59	101	63.10
19: 00–23–59	35	21.90
00: 00–6–59	24	15.00
**Cardiac functional grading**		
Grade I	112	70.00
Grade II	36	22.50
Grade III	9	5.60
Grade IV	3	1.90
**Smoke**		
No	94	58.80
Yes	66	41.30
**Alcoholic drinks**		
No	134	83.80
Yes	26	16.30
**Degree of chest pain**		
Painless (0)	13	8.10
Mild pain (1–3)	7	4.40
Moderate pain (4–6)	20	12.50
Severe pain (7–10)	120	75.00
**Self - rescue**		
No	136	85.00
Yes	24	15.00
**Family history of coronary heart disease**		
No	158	98.80
Yes	2	1.30
**Chronic comorbidities**		
No	43	26.90
Yes	117	73.10
**Hypertension**		
No	30	18.80
Yes	85	53.10
**Hyperlipidemia**		
No	86	53.80
Yes	29	18.10
**Diabetes**		
No	62	38.80
Yes	53	33.10
**Others**		
No	102	63.70
Yes	15	9.40

### Fear of progression in AMI patients

In this study, the total score of fear of progression in 160 patients with AMI was (33.43 ± 7.09), among which the score of fear in the physical health dimension was (16.67 ± 3.64), and the score of fear in the social and family dimension was (16.76 ± 3.92), and the score of fear in the social and family dimension was roughly the same as that in the physical health dimension. Among the items of the simplified the Fear of Progression Questionnaire- Short Form, item 9 " I fear that there will be some major treatment in the course of the disease” had the highest score (3.13 ± 0.88); This was followed by item 4, " The thought of being less productive because of illness annoys me” (3.04 ± 0.91) and item 11, " I worry about what will happen to my family if something happens to me” (3.04 ± 0.82). Among the 160 patients with AMI, 81 cases (50.60%) had the disorder of fear of progression (Tables 3, 4 and 5).

**Table 3:** Fear of progression scores in patients with AMI

Variables	Scoring range	Minimum	Maximum	Score
Fear in the socio-family dimension	1–5	6	30	16.76 ± 3.92
Fear in physiological health dimension	1–5	6	29	16.67 ± 3.64
Fear of progression	12–60	12	59	33.43 ± 7.09

**Table 4:** Scores of each item of the fear of Progression Scale in patients with acute myocardial infarction

Variables	Scoring range	Minimum	Maximum	Score
1. I became anxious at the thought of the disease progressing.	1–5	1	5	2.96 ± 0.92
2. I feel nervous before a doctor’s exam and some regular medical check-ups.	1–5	1	5	2.28 ± 0.81
3. I’m afraid of the pain caused by this disease.	1–5	1	5	2.94 ± 0.87
4. The thought of being less productive because of illness annoys me.	1–5	1	5	3.04 ± 0.91
5. I have some physical discomfort when I am anxious (e.g., rapid heartbeat, stomach pain, nervousness, etc.).	1–5	1	5	2.49 ± 0.70
6. I’m worried that my disease might be passed on to my children.	1–5	1	5	2.39 ± 0.69
7. The possibility of having to rely on strangers in my daily life makes me anxious.	1–5	1	5	2.76 ± 0.77
8. I worry that I will not be able to continue my hobbies/hobbies at some point due to illness.	1–5	1	5	2.53 ± 0.67
9. I fear that there will be some major treatment in the course of the disease.	1–5	1	5	3.13 ± 0.88
10. I’m afraid the drugs will damage my body.	1–5	1	5	2.87 ± 0.95
11. I worry about what will happen to my family if something happens to me.	1–5	1	5	3.04 ± 0.82
12. The idea that I might not be able to work because of illness bothers me.	1–5	1	5	2.99 ± 0.93

**Table 5:** Dysfunctions of fear of progression in patients with AMI

Degree of fear of progression	*n*	Percentage(%)
The fear of progression is dysfunctional (FoP score ≥ 34)	81	50.60
The fear of progression is not dysfunctional (FoP score < 34)	79	49.40

### Influencing factors of fear of progression in patients with AMI

To examine the demographic characteristics and disease-related characteristics that might influence the total score of fear of disease progression in AMI patients, a T-test and one-way analysis of variance were used. The results showed that gender, monthly income, education level and chest pain degree were the factors affecting the total score of fear of disease progression in AMI patients (*p* < 0.05). Among them, the score of fear of progression in women was significantly higher than that in men, and the score of fear of progression in patients with monthly income below 1000 yuan was significantly higher than that in patients with monthly income above 5000 yuan. Patients with primary school education had significantly higher scores of fear of progression than those with middle school education, high school education, college education and above. In terms of the degree of chest pain, patients who reported severe pain had significantly higher scores of fear of progression than patients who reported no pain and patients with mild pain (Table 6).

**Table 6:** Fear of disease progression by demographic profile

Variables	Total fear of progression(Mean ± SD)	t/F-value	*P*
**Gender**			
Male (*n* = 123)	32.21 ± 6.67	-4.142	<0.001*
Female (*n* = 37)	37.46 ± 7.05		
**Age(years)**			
<45 (*n* = 25)	33.68 ± 7.07	0.439	0.645
45 ~ 60 (*n* = 60)	32.75 ± 7.14		
>60 (*n* = 75)	33.88 ± 7.11		
**Marital status**			
Married (*n* = 148)	33.66 ± 7.07	0.900	0.443
Spinsterhood (*n* = 5)	32.20 ± 6.26		
Divorce/separated (*n* = 5)	29.40 ± 8.02		
widowed (*n* = 2)	29.00 ± 9.90		
**Employment**			
Employed (*n* = 40)	32.85 ± 6.45	0.152	0.929
No job/resignation (*n* = 60)	33.70 ± 7.32		
Retired (*n* = 41)	33.34 ± 5.96		
Farmer (*n* = 19)	33.95 ± 9.91		
**Monthly income**			
≤ 1000 yuan (*n* = 48)	35.56 ± 8.38	2.920	0.036*
1000–3000 yuan (*n* = 60)	32.97 ± 6.43		
3000–5000 yuan (*n* = 39)	32.72 ± 6.07		
≥ 5000 yuan (*n* = 13)	29.77 ± 6.02		
**Education**			
Primary and below (*n* = 25)	37.28 ± 7.45	3.704	0.013*
Junior high school (*n* = 60)	33.53 ± 7.67		
Senior high school (*n* = 63)	32.27 ± 6.36		
University and above (*n* = 12)	30.92 ± 3.75		
**Payment**			
Medical insurance (*n* = 153)	33.47 ± 7.09	0.38	0.705
Self-financed or other (*n* = 7)	32.43 ± 7.63		
**Type of AMI**			
STE-MI (*n* = 97)	33.04 ± 6.63	-0.849	0.397
Non-st elevation type (*n* = 63)	34.02 ± 7.76		
**Number of coronary artery infarcts**			
Single lesion (*n* = 90)	33.99 ± 7.59	1.141	0.255
Multiple lesion (*n* = 70)	32.70 ± 6.38		
**Treatment method**			
Drug Conservative therapeutic (*n* = 32)	34.84 ± 9.13	1.035	0.307
Interventional Surgery (*n* = 128)	33.07 ± 6.48		
**Onset time**			
7: 00–18–59 (*n* = 101)	33.34 ± 6.74	0.362	0.697
19: 00–23–59 (*n* = 35)	32.94 ± 1.16		
00: 00–6–59 (*n* = 24)	34.50 ± 1.81		
**Cardiac functional grading**			
Grade I (*n* = 112)	33.17 ± 0.69	1.318	0.271
Grade II (*n* = 36)	34.47 ± 1.07		
Grade III (*n* = 9)	30.67 ± 2.25		
Grade IV (*n* = 3)	38.67 ± 3.38		
**Smoke**			
No (*n* = 94)	34.21 ± 7.11	1.686	0.094
Yes (*n* = 66)	32.30 ± 6.97		
**Drink**			
No (*n* = 134)	33.70 ± 7.28	1.12	0.264
Yes (*n* = 26)	32.00 ± 5.93		
**Degree of chest pain**			
Painless (*n* = 13)	29.77 ± 6.61	3.58	0.015*
Mild pain (*n* = 7)	27.86 ± 5.93		
Moderate pain (*n* = 20)	32.30 ± 5.53		
Severe pain (*n* = 120)	34.33 ± 7.20		
**Self - rescue**			
No (*n* = 136)	33.35 ± 6.73	-0.305	0.761
Yes (*n* = 24)	33.83 ± 9.04		
**Family history of coronary heart disease**			
No (*n* = 158)	33.34 ± 7.09	-1.424	0.156
Yes (*n* = 2)	40.50 ± 3.54		
**Chronic comorbidities**			
No (*n* = 43)	32.67 ± 7.41	-0.811	0.419
Yes (*n* = 117)	33.70 ± 6.99		

Abbreviations: *p<0.05

Pearson correlation analysis showed that the fear of progression and its dimensions were positively correlated with the resignation (*r* = 0.644 ~ 0.702) and avoidance (*r* = 0.499 ~ 0.547) dimensions of medical coping styles, but not with the confronce dimensions. There was a positive correlation with disease perception, and both were statistically significant (*r* = 0.698 ~ 0.763, *P* < 0.01). It was negatively correlated with the total score of psychological capital (*r*= -0.668~-0.719) and the four dimensions of self-efficacy (*r*= -0.534~-0.598), optimism (*r*= -0.549~-0.622), hope (*r*= -0.589~-0.658) and resilience (*r*= -0.685~-0.739), and all had statistical significance (*r*= -0.534~-0.739, *P* < 0.01). It was negatively correlated with the total score of social support, objective social support, subjective social support and social support utilization, and all had statistical significance (*P* < 0.01) (Table 7).

**Table 7:** Correlation analysis between fear of progression and medical coping style, disease perception, psychological capital and social support in AMI patients

Variables	Physiological health dimension	Socio-family dimension	Fear of progression
**Medical coping style**			
Confronce	0.115	-0.039	0.038
Avoidance	0.528**	0.499**	0.547**
Resignation	0.674**	0.644**	0.702**
**Disease perception**	0.734**	0.698**	0.763**
**Psychological capital**	-0.680**	-0.668**	-0.719**
Self-efficacy	-0.534**	-0.586**	-0.598**
Optimistic	-0.619**	-0.549**	-0.622**
Hope	-0.648**	-0.589**	-0.658**
Resilience	-0.685**	-0.701**	-0.739**
**Social support**	-0.482**	-0.461**	-0.503**
Objective social support	-0.370**	-0.283**	-0.346**
Subjective social support	-0.372**	-0.413**	-0.420**
Social support availability	-0.408**	-0.358**	-0.408**

Abbreviations: ** At level 0.01 (two-tailed), the correlation was significant

In order to further clarify the effects of variables in general data and related variables on the fear of progression in AMI patients, the total score of fear of progression was taken as the dependent variable, and statistically significant variables in univariate analysis and correlation analysis were taken as independent variables. Multiple linear regression analysis was performed, including gender, monthly income, educational level, the degree of chest pain, social support, psychological capital, disease perception, resignation and avoidance. *P* < 0.05 was taken as the test standard. The assignment of the independent variables was as follows: Gender “male = 1, female = 2”; monthly income “≤1000 = 1, 1000–3000 = 2, 3000–5000 = 3, ≥ 5000 = 4”; Education “Primary and below = 1, Junior high school = 2, Senior high school = 3, University and above = 4”; Degree of chest pain “Painless = 1, Mild pain = 2, Moderate pain = 3, Severe pain = 4”; Disease perception, social support, psychological capital and avoidance are the actual measured value. According to the Tolerance and Variance inflation factor (VIF) in the multicollinearity test method, it is usually required that VIF < 10 or Tolerance ≥ 0.1 to determine whether the independent variable has collinearity. It shows that there is no serious multicollinearity between the independent variables. The VIF values in this study are all less than 5, indicating that there is no multicollinearity problem among the independent variables in this study. The results showed that gender, disease perception, psychological capital and resignation were the factors influencing the fear of disease progression in AMI patients, accounting for 67.4% of the total variation (Table 8).

**Table 8:** Multi-factor analysis of fear of progression in acute myocardial infarction patients

Variables	Unstandardized coefficient		Standardized Coefficient	T-vaule	*P*-vaule	Collinearity Statistics	
	B	Standard error	Beta			Tolerance	VIF
(Constant)	12.45	6.81	-	1.828	0.07		
Gender	1.971	0.804	0.118	2.452	0.015	0.892	1.121
Monthly income	0.265	0.474	0.035	0.559	0.577	0.533	1.877
Education	0.189	0.534	0.022	0.354	0.724	0.513	1.948
Degree of chest pain	0.017	0.381	0.002	0.045	0.964	0.859	1.164
Disease perception	0.325	0.051	0.445	6.408	< 0.001	0.424	2.357
Social support	0.09	0.086	0.068	1.051	0.295	0.496	2.014
Psychological capital	-0.095	0.031	-0.252	-3.011	0.003	0.293	3.41
Avoidance	0.526	0.274	0.117	1.921	0.057	0.554	1.806
Resignation	0.439	0.203	0.167	2.159	0.032	0.343	2.914
Adjusted R^2^				0.674			
*F*				37.526			
*P*				< 0.001			

## Discussion

### FoP in AMI patients is moderate

This study focused on investigating the degree of fear of progression in patients with acute myocardial infarction and the related factors affecting their fear of progression, which is not only a supplement to the research on the related factors affecting fear of progression in patients with acute myocardial infarction, but also provides important insights for medical personnel to help patients with acute myocardial infarction develop intervention measures to reduce their fear of progression and promote their recovery. In this study, the overall fear of progression was at a moderate level, which was related to gender, disease perception, medical coping style of resignation and psychological capital.

Contradicting the study of Gao Yanli and Su Jin, the results of this study showed that the total score of FoP level in AMI patients was higher than that in Gao Yanli’s study of FoP level in AMI patients [18], and lower than that in Su Jin’s survey of FoP level in young and middle-aged AMI patients [40]. The reason may be attributed to the fact that the patients in this study were younger than patients admitted in study of Gao Yanli [18]. At present, studies have confirmed that the younger the patient, the higher the FoP level [41–43], which is because, on the one hand, the younger the patient, the lower the patient’s ability to cope with stressful events, and the ability to overcome fear [44]. On the other hand, because younger patients tend to bear a heavier financial burden in the family, there is a higher level of fear of progression. In addition, middle-aged and young patients are often in the development period of their careers at this time, and the arrival of the disease will also disrupt their career planning and aspirations. The results of this study also showed that the FoP level of AMI patients was correlated with gender, and the FoP level of female AMI patients was higher than that of male patients, which was consistent with the survey results conducted by Gu Jiayun [45] and Wu Qiyun [46] on the FOP level of inpatients with primary liver cancer and their spouses in the “gap period” after radiofrequency ablation of atrial fibrillation patients. It is suggested that medical workers should formulate personalized treatment and nursing methods according to different conditions and individual needs of patients [47–49], and provide health education for acute myocardial infarction. At the same time, they should pay attention to the progress of return to work and psychological state of patients after their condition is stabilized, provide vocational assistance channels in time and strengthen psychological counseling [40].

### Analysis of influencing factors of FoP in AMI patients

#### The higher the illness perception of AMI patients, the higher the FoP level

In addition, the overall disease perception score of AMI patients was above the medium level, which was positively correlated with the FoP of AMI patients. This is consistent with the FoP survey results of Gu Jiayun [45], Lin Weitong [50], Shi Le [51], Liu [52] and Shi Xianjun [53] in atrial fibrillation patients with “gap period” after radiofrequency ablation, kidney transplant patients, nephrotic syndrome patients and interstitial lung disease patients, and young and middle-aged patients after percutaneous coronary intervention, respectively. However, the higher the patients’ disease perception, the more serious their negative perception will be, thus taking a negative coping style to the treatment of AMI, which will further affect the patients’ recovery [54]. In this regard, it is suggested that medical workers provide AMI knowledge and health education and personalized psychological guidance to patients to improve their cognition of AMI. Furthermore, patients can be helped to establish a positive disease perception and coping style, cooperate with the treatment of medical workers, improve the level of patients’ cardiac function, prevent the recurrence of myocardial infarction, and reduce the rate of re-hospitalization [54–56].

#### The higher the psychological capital of AMI patients, the lower the level of FoP

In addition, the total score of psychological capital and its dimensions of AMI patients were found to be negatively correlated with FoP, which was consistent with the research of Dong Jianqing, Tian Ruxiang and Wang Jiajia [57–59]. Studies have pointed out that psychological capital has a strong mediating effect between patients’ perceived stress level and FoP [57]. The higher the positive psychological capital of patients, the higher their quality of life [60, 61]. By improving the level of psychological capital of patients, patients’ fear of progression or relapse can be reduced, thus reducing the impact of adverse emotions [62, 63]. Thus, patients have a higher confidence in the recovery of the disease and improve the compliance and health literacy of patients receiving treatment [64]. In this study, the psychological capital of AMI patients was at a moderate level. Therefore, medical workers should timely evaluate the psychological state of patients, help them relieve anxiety, and carry out targeted psychological intervention through painting therapy, psychological cognitive therapy, acceptance and commitment therapy [62] to improve the level of psychological capital and FoP of patients and enhance their confidence in disease control.

#### The level of FoP was higher in AMI patients with resignation coping style

The results of this study showed that the resignation coping style was positively correlated with FoP, which was consistent with the FoP levels investigated by Cao Ningning [65], Niu Rui [66], Ding Sicheng [67] and Tang Qingyun [68] in patients with type 2 diabetes mellitus, patients with systemic lupus erythematosus, patients with advanced gastric cancer undergoing postoperative chemotherapy and young men who have sex with men with HIV/AIDS. Resignation means that patients neither suppress their negative feelings nor take measures to improve their negative emotions, allowing their negative emotions to grow, resulting in further aggravation of their negative psychology [69]. Patients who adopt a negative coping style will negatively evaluate themselves and the treatment of the disease, resulting in pessimism and negative cooperation with treatment, which is not conducive to physical and mental rehabilitation [65]. Studies have shown that patients’ FoP psychology is affected by negative coping styles, and the more negative coping, the stronger the patients’ fear of progression [18, 65]. Among them, 72.6% of patients are complicated with chronic diseases such as hypertension and diabetes. Pathological states of various diseases increase the incidence of adverse events in AMI patients [20], increase the physical and mental pressure of patients, and lead them to face the rehabilitation of diseases with negative emotions, thus choosing the coping methods of compromise and resignation. Therefore, medical workers should pay attention to the psychological state of patients, identify and correct patients’ surrender coping styles in time, help patients correctly understand the disease through targeted disease knowledge and health guidance, encourage and guide patients to adopt positive coping ways to face the treatment and rehabilitation process of their diseases, and improve the level of FoP.

#### The less social support of AMI patients, the higher the level of FoP

Good social support can help patients cope with sudden stress events, help patients maintain stable emotions, and play an important role in the improvement of patients’ physical and mental health. Studies have shown that less social support for patients can lead them to avoid or solve any problems on their own to cope with the disease, which amplifies the perception of the impact of the disease on the patient’s life, thereby increasing the psychological stress and feelings of the patient, and thus producing excessive FoP [70]. In this study, the correlation analysis showed that the less social support of AMI patients, the higher the level of FoP, while the multivariate regression analysis did not show this result, which may be related to the small sample size and the limitation of one hospital in this study. In the future, the sample size should be expanded to further verify the impact of social support.

#### Limitations and recommendations

There are still the following shortcomings in this study. All subjects were recruited in the cardiology department of a Grade-III hospital in Changchun, and the sample sources had certain geographical limitations, and the sample representation was insufficient. The survey time was after AMI patients were admitted to hospital and their condition was stable, and this study was a cross-sectional survey, and the results could only reflect the progression of disease fear in the early stage of patients’ condition stabilization. In the future, multi-center longitudinal studies could be conducted to further understand the trajectory of progression and influencing factors of AMI patients.

## Conclusions

Through cross-sectional investigation, this study found that the fear of progression in AIM patients was above the medium level, and gender, disease perception, psychological capital and resignation were the influencing factors of the fear of progression in AMI patients. Nursing staff should focus on AIM patients suffering from multiple diseases and lack of awareness of their own diseases, and carefully observe the psychological status of patients, personalized knowledge health guidance and psychological nursing, in order to improve the recovery of patients, quality of life and FoP level. In addition, studies on the effects of pain, quality of life, adherence to treatment, interventional surgery, and medication on FoP in patients with AMI are recommended.

## Data Availability

The datasets used and/or analyzed in the current study are available from the corresponding author’s email upon reasonable request.

## Abbreviations

AMI: Acute myocardial infarction
FoP: Fear of progression
